# 

**Published:** 1908-02

**Authors:** 


### BOOKS RECEIVED.

Transactions of the Fifth Annual Conference of State and Territorial Health Officers, with the United States Public Health and Marine Hospital Service. Held at Washington, D. C., May 29, 1907. Washington: Government Printing Office.

The Practical Medicine Series of Year Books. Ten volumes. Issued under the general editorial charge of Gustavus P. Head, M.D., Professor of Laryngology and Rhinology in the Chicago Post-Graduate Medical School. Vol. X. Nervous and Mental Diseases. Series 1907. Chicago: The Year Book Publishers. (Price, $1.25, entire series, $10.00.)

Cosmetic Surgery. The correction of featural imperfections. By Charles C. Miller, M.D. 136 pages. 73 illustrations. Published by the Author, 70 State St., Chicago, Ill. (Cloth, $1.50.)

Diseases of the Nose and Throat. By D. Braden Kyle, M.D., Professor of Laryngology and Rhinology, Jefferson Medical College, Philadelphia. Fourth Edition. Octavo, 725 pages, with 215 illustrations, 28 in colors. Philadelphia and London: W. B. Saunders Company, 1907. (Cloth, $4.00; half morocco, $5.50 net prices.)

Atlas and Textbook of Human Anatomy. Volume III. By Professor J. Sobotta, of Wurzburg. Edited, with additions, by J. Playfair McMurrich, A.M., Ph.D., Professor of Anatomy at the University of Michigan, Ann Arbor. Quarto, 342 pages, containing 297 illustrations, mostly in colors. Philadelphia and London: W. B. Saunders Company. 1907. (Cloth, $6.00; half morocco, $7.00, net prices.)

A Textbook of Minor Surgery. By Edward Milton Foote, A.M., M.D., Instructor in Surgery in the College of Physicians and Surgeons (Columbia University) New York. Octavo, pp. 778. Illustrated by 407 engravings from original drawings and photographs. New York and London: D. Appleton and Company. 1908. (Cloth, $5.)

Gonorrhea, its Diagnosis and Treatment. By Frederick Baumann, Ph.D., M.D., Professor of Genitourinary Diseases in the Reliance Medical College, Chicago. 12 mo, pp. 218. Illustrated. New York and London: D. Appleton and Company. 1908. (Cloth, $1.50.)

Transactions of the twenty-third annual meeting of the Oregon State Medical Association held at Seaside, Ore., July 12, and 13, 1907. L. H. Hamilton, M.D., Secretary.

The Medical and Surgical Knowledge of William Shakspere. With Explanatory Notes. By John W. Wainwright, M.D. Octavo, pp. 78. New York: Published by the author. 1907.

Essentials of Modern Electrotherapeutics. An Elementary Textbook on the Scientific Therapeutic Use of Electricity and Radiant

Energy. By Frederick Finch Strong, M.D. Instructor in Electro-therapeutics at Tufts College Medical School, Boston. New York: Rebman Company. (Price, $1.00).

